# Quantifying the effectiveness and health co-benefits of climate change mitigation actions across sectors: a protocol for an umbrella review

**DOI:** 10.12688/wellcomeopenres.17498.2

**Published:** 2022-08-25

**Authors:** Kristine Belesova, Rosemary Green, Romain Clercq-Roques, Jane Falconer, Hugh Sharma Waddington, Sarah Whitmee, Tamzin Reynolds, Syreen Hassan, Andy Haines

**Affiliations:** 1 Department of Public Health, Environments and Society and Centre on Climate Change and Planetary Health, London School of Hygiene and Tropical Medicine, London, WC1H 9SH, UK; 2 Department of Population Health and Centre on Climate Change and Planetary Health, London School of Hygiene and Tropical Medicine, London, WC1E 7HT, UK; 3 Library, Archive & Open Research Services, London School of Hygiene and Tropical Medicine, London, WC1E 7HT, UK; 4 Environmental Health Group, Disease Control Department, London School of Hygiene & Tropical Medicine, London International Development Centre, London, WC1A 2NS, UK

**Keywords:** climate change, mitigation, climate action, actions, planetary health, health, systematic review, evidence synthesis, umbrella review

## Abstract

Background: Effective and rapid actions are required to achieve global goals for climate change mitigation, and there is an opportunity to ensure that the actions taken are also positive for human health. However, little is known about the relative magnitude of the health co-benefits that can be achieved from mitigation actions, so robust and comprehensive syntheses of the evidence on the nature and effects of relevant actions are required. This paper presents a protocol for an interdisciplinary and cross-sectoral umbrella review of systematic reviews, synthesising modelled and empirical evidence on such actions.

Methods: Nine bibliographic databases will be searched, capturing literature across a wide range of disciplines and sectors. Unique records retrieved by the searches will be screened by two independent reviewers. The quality of all the included systematic reviews will be assessed using A MeaSurement Tool to Assess Systematic Reviews (AMSTAR) 2 critical appraisal tool. Data will be extracted on methodological and thematic characteristics of the reviews, nature of the actions, and their effects on greenhouse gas emission reduction, health, and its determinants, as well as any other reported effects and interactions across different actions.

Results: Narrative and quantitative synthesis methods will be used to create a typology of relevant actions, map pathways to their impacts on health, compare the magnitude of health and greenhouse gas (GHG) emission reduction impacts by selected characteristics of the actions and the nature of the evidence, as well as to identify gaps in evidence syntheses.

Conclusion: This review will identify the most effective actions for global climate change mitigation and health based on the best available scientific evidence.

This protocol has been registered in PROSPERO, Reg No.: CRD42021239292.

## Introduction

The Paris Agreement’s goal to limit global average temperature increase to well below 2°C, preferably to 1.5°C, compared to pre-industrial levels requires reaching net zero greenhouse gas emissions globally by 2050 if not earlier ( United Nations Environment Programme, 2019). Our society is, therefore, facing the responsibility to identify and implement actions capable of triggering and sustaining system-wide transitions from the current greenhouse gas (GHG) emission trajectory onto a pathway for timely achievement of the net zero target. To meet all three dimensions of sustainable development – social, economic, and environmental – such a trajectory should allow everyone to thrive within the planetary boundaries ( Steffen *et al.*, 2015; Whitmee *et al.*, 2015). It has been estimated that millions of premature deaths could be averted by reducing air pollution through fossil fuel phase out, transitioning to healthy and sustainable diets and housing, developing net zero health care systems, increasing the proportion of active travel and use of active transport, and implementing nature-based solutions, including reforestation and reducing deforestation ( Aleksandrowicz *et al.*, 2016; IUCN, 2016; Lelieveld *et al.*, 2019; Lenzen *et al.*, 2020; Pencheon & Wight, 2020; Seddon *et al.*, 2021; Wilkinson *et al.*, 2009; Willett *et al.*, 2019). Therefore, priority must be given to identifying and implementing actions that not only offer the greatest climate change mitigation potential, but also have significant potential to positively contribute to health worldwide and avoid adversely affecting health outcomes (e.g., through regressive policies). 

To accelerate the necessary system-wide transitions and implementation of the most promising actions, there is an urgent need for a comprehensive interdisciplinary and cross-sectoral synthesis of evidence on the range of actions available and their effectiveness in mitigating climate change and improving human health and wellbeing. The synthesis should also account for any evidence of unintended consequences and spillover effects (i.e., positive or negative effects of one actor’s mitigation actions on another actor’s ability to achieve mitigation or other sustainable development objectives), synergies and trade-offs, as well as implications for health equity. To date, many published systematic reviews have focussed on a single sector, discipline, and/or category of impact; for example, only climate change mitigation impact or health impact and vice versa (e.g., Cantzler *et al.*, 2020; Court & Sorrell, 2020; Hertwich *et al.*, 2019; Javaid *et al.*, 2020; Sethi *et al.*, 2020). Such focussed reviews often offer limited opportunities to understand the cross-sectoral, cross-disciplinary, and system-wide implications of climate change mitigation actions, reducing our capacity to draw balanced conclusions on the most effective actions and their synergistic combinations. Furthermore, most systematic reviews do not attempt quantitative synthesis of the impact of actions on greenhouse gas emissions and health, as the actions and designs of the original studies that quantify their impact are highly heterogenous.

The Pathfinder Initiative aims to identify and synthesise evidence on actions that accelerate progress towards a zero-carbon economy and simultaneously help to improve health locally and globally. Although the focus of the initiative is on climate change mitigation, the adaptation actions are in scope of our work as long as they also have a significant mitigation component. The Pathfinder Initiative will provide evidence on the important gaps in knowledge on which actions can help achieving the greatest benefits with minimal trade-offs for the climate and health in different contexts and how these actions can be effectively scaled-up ( Hassan *et al.*, 2021).

Here we report a protocol for an umbrella review – a review of systematic reviews or meta-analyses, undertaken as a part of the Pathfinder Initiative, to synthesise the existing syntheses of findings on the range and effectiveness of actions – both potential and already implemented in practice – for climate change mitigation and health across all sectors and disciplines that can help triggering and sustaining the necessary system-wide transitions. We focus not only on the short-term health benefits of climate change mitigation actions but also on the health benefits that would occur as a result of climate change mitigation in the long term by reducing the anticipated future health impacts of climate change. The umbrella review will compare results across sectors and disciplines and provide synthesis of any inter-sectoral and inter-scalar effects (i.e., the effects of actions implemented in one sector or scale on other sectors or scales), including synergies and trade-offs. Modelled or observed evidence on unintended consequences, spillover effects, and distributional effects with implications for health equity will also be synthesised where available. We will assess the nature and quality of the existing systematic evidence syntheses and map the ‘absolute gaps’ in evidence that require new primary studies, ‘synthesis gaps’ that require new focussed systematic reviews and syntheses, and ‘synthesis update gaps’ where existing reviews are based on outdated searches and updates of the existing focussed reviews are necessary. 

### Aim and research questions

The aim of the umbrella review is to assess and synthesise the nature, quality, and strength of the systematically reviewed evidence on climate change mitigation actions (and their health co-effects, which largely are co-benefits but may involve certain trade-offs) for a range of system wide transitions and their corresponding sectors including but not limited to, energy supply, transport, buildings, industry, agriculture, forestry, and other land use (AFOLU), human settlements/infrastructure, and nature-based actions. We will focus on addressing the following research questions:

1. What types of climate change mitigation actions with defined short-term and long-term health co-benefits have been reported in systematic reviews and what are their pathways of impact on health?
2. How does the magnitude of change in health outcome or exposure and GHG emissions differ within and between the identified types of actions, based on the modelled as compared to the observational evidence (provided there is sufficient observational information of implemented actions), and across different contexts?
3. How do syntheses differ in their coverage, quality, and strength of evidence across sectors, disciplines, action types, and their implementation or modelling contexts? Are there any sectors where no syntheses exist?

## Protocol

### Inclusion criteria

The scope of our umbrella review is defined by the following inclusion criteria of existing systematic reviews.

**Population**: studies with exposures and impacts of relevance to human population without geographical restrictions

**Intervention**: actions primarily targeting climate change mitigation or known to have an impact on climate change mitigation alongside other primary targets, e.g., improving health and climate change adaptation. We will include interventions undertaken in any sector, broadly: Energy systems; Agriculture, Forest and Other Land Use (AFOLU); Transport; Buildings; Industry; and Land, Coastal and Ocean Sinks, and Engineered Sinks. These could be single solutions carried out within single sectors, multiple actions carried out in single sectors (e.g., system transitions), or multiple actions carried out across multiple sectors. Several classification systems exist and could be used to group and present our findings, for example, the classification system used in the IPCC AR6 WG3 report (Figure SPM.8) ( IPCC, 2022), or the Drawdown Framework for Climate Solutions (Project Drawdown, 2022). We will use and if necessary, adapt the classification system to which our data fits best.

**Outcome**:
  (1) any form of documented changes in health outcomes (e.g., all-cause or cause-specific mortality, years of life lost, morbidity from respiratory, cardiovascular and other diseases) or its well-established determinants whether positive or negative, short-term or long-term (i.e., as a result of reducing potential future health impacts of climate change), including changes in those exposures that are known to increase the risk of adverse health outcomes, e.g., environmental risk factors included in the Global Burden of Disease Assessment ( Abbafati *et al.*, 2020). The preliminary list of eligible outcomes, exposures, and risk factors is available in the Table 1
  (2) change in emissions or atmospheric concentrations of major greenhouse gases: Carbon dioxide (CO _2_), Nitrous oxide (N _2_O), Methane (CH _4_), or short-lived climate pollutants such as black carbon
**Study design**: systematic review, defined as a study that identifies explicitly as a systematic review and meets the Preferred Reporting Items for Systematic Reviews and Meta-analysis (PRISMA) criteria, including an explicit systematic search strategy and clear criteria for the inclusion of original studies that report the impact of actions on both outcomes outlined above, synthesised using meta-analysis or narrative synthesis approaches
**Year of publication:** from 2000 until present to capture reviews published after the introduction of the Millennium Development Goals (MDGs), wider acceptance of the concept of health co-benefits, and the establishment of international bodies coordinating high quality reviews on social and environmental topics (e.g., Campbell Collaboration founded in 2000, Collaboration for Environmental Evidence founded in 2006)
**Language:** studies published in English, Spanish, French, Portuguese, Chinese, Russian, German, Dutch, Italian, Arabic

**Table 1.** The preliminary list of health outcomes and exposures/risk factors * that will be used to guide inclusion and exclusion as well as assist the extraction of data on health outcome/exposure types. Adapted from Table 5 from Hassan *et al.* (2021), available under a CC-BY-4 license.

Health outcomes
Disease manifestations of poor diets (vitamin and mineral deficiencies) Disease manifestations of air pollution (chronic lung and cardiovascular diseases) Disease manifestations of increased climate sensitive pathogens (malaria, Lyme disease, West Nile virus, enteric diseases) Weather-related morbidity and mortality with implications for productivity Transport related morbidity/mortality (road traffic accidents) Mental health conditions arising from climate-related exposures Morbidity and mortality related to the urban heat island effect
Risk factors for ill health (based on the risk factors used in the Global Burden of Disease project ( Abbafati *et al.*, 2020))
Childhood underweight and overweight Diet low in fruits, vegetables, legumes, nuts and seeds, seafood and omega-3 fatty acids Diet high in red meats Overweight and obesity (BMI) Physical inactivity Ambient particulate matter and ozone pollution Household air pollution exposure Noise pollution Chemical pollution Poor housing insulation leading to cold indoor conditions Crowding / physical proximity Exposures to climate change impacts Disruptions to water supply and quality Disruptions to energy Disruptions to healthcare access Disruptions to food supply Displacement Increase in climate sensitive pathogens and vectors (ticks, mosquitoes, sand flies)
Socio-economic determinants of health
Poverty Homelessness Unemployment Female education and female participation in the workforce Governance and decision-making processes

*We include both short-term health co-benefits of climate change mitigation as well as long-term impacts of climate change on health that can be avoided through climate change mitigation.

To develop our methodology, we reviewed some of the relevant systematic reviews (that would be included in our analyses) and concluded that the data reported in the systematic reviews often falls short from the level of detail required to address the objectives of our umbrella review. For example, many systematic reviews are narrative and do not attempt any quantitative synthesis even when such synthesis would be feasible. Furthermore, systematic reviews without a quantitative synthesis often do not report sufficient data from the primary studies that they reviewed, e.g., only reporting either the health or GHG impact of the actions but not both or lacking information on the baseline or scale of the impact, which are necessary to allow comparing the impact of actions across studies and settings. However, these data are often available from the text of the primary studies. Therefore, we adapted the umbrella review methodology to expand our data extraction procedure beyond the (often limited) text of the systematic reviews to the text of primary studies that were included in these systematic reviews. We propose to extract data from relevant primary studies included in eligible systematic reviews to capture the data on action health and GHG emission impact estimates and their characteristics that are not reported in the systematic reviews. We will use the following criteria to select eligible primary studies:

**Study designs:** any experimental or observational (including natural experimental) empirical study or a modelling study that reports quantitative estimates using the following methods:
  (1) randomised controlled trials (RCTs) using random assignment of units to groups with and without the mitigation action, and quasi-RCTs using prospective methods of assignment of units such as alternation
  (2) studies with non-random assignment of units to mitigation actions and comparison with pre-test and post-test data collection (controlled before *vs* after), or studies with post-test data collection only (cross-sectional and case-control studies)
  (3) studies with single group data and sufficient observations to establish trends before and after a mitigation action, i.e., interrupted time series (ITS), and ITS incorporating a control group (controlled-ITS)
  (4) non-randomised studies with assignment to a mitigation action based on a threshold on an ordinal or continuous assignment variable (regression discontinuity design), or a geographical or administrative boundary (geographical discontinuity design), comparing outcomes for groups around the threshold
  (5) non-randomised studies, sometimes called natural experiments, using statistical approaches to account for unobservable confounding (e.g., difference-in-differences, instrumental variables)
  (6) modelling studies are eligible where they compare a scenario with a mitigation action against a business as usual or another scenario without a mitigation action, and have modelled GHG emissions and health impacts (including impacts on exposures and health determinants) of the scenarios
**Comparisons:** eligible comparisons include ‘no mitigation action’, a ‘wait-list’ where subjects/places that have not yet been exposed to the mitigation action are used as controls, ‘business as usual’ or another strategy that does not lead to reductions in GHG emission or atmospheric concentrations. Studies with active controls, i.e., a comparison of two mitigation action scenarios (without a scenario where there are no mitigation actions), are excluded.

This protocol has been registered in PROSPERO, Reg No.: CRD42021239292 ( Belesova *et al.*, 2021b).

### Literature search

Our searches will be performed in the following nine databases: Medline ALL, Embase, Global Health and Econlit on the OvidSP platform; Africa-Wide Information and GreenFILE on the Ebsco platform and Web of Science Core Collection, BIOSIS Citation Index and SciELO on the Clarivate Analytics Web of Science platform. The structure of our search consists of two search blocks combined as follows: (climate change mitigation terms) AND (systematic review terms). The search strategy was developed by a librarian experienced in systematic review borrowing climate change and climate change mitigation search terms from the search strategy developed by Belesova *et al.* (2021a) and Falconer (2014) and terms for reviews from Lee *et al.* (2012). The search terms were reviewed by members of the Pathfinder Initiative, implementing their recommendations where appropriate. The search syntax was adapted for each of the bibliographic databases. The full search strategy for each database is available through the LSHTM Data Compass depository ( Belesova *et al.*, 2022). In addition, to ensure coverage of peer-reviewed systematic reviews that remain outside of academic databases, we will hand search the libraries of the Campbell Collaboration, Collaboration for Environmental Evidence, and the EPPI-centre.

### Screening for inclusion

Screening for inclusion will follow a two-stage approach. In the first stage, the titles and abstracts will be screened against the inclusion criteria and in the second – full texts. The unique records identified from our searches will be first downloaded into the Rayyan open access software where the title and abstract screening will be performed ( Ouzzani *et al.*, 2016). All records eligible for the full text review will be then imported into EPPI-Reviewer software where the full text screening, quality assessment, and data extraction will be performed ( Thomas *et al.*, 2010). We will use the PRISMA statement and flow diagram to record the search and screening results. We will record all reasons for exclusion at the full text review level. In all stages, screening will be performed by pairs of independent reviewers, who will reconcile any differences, consulting a third reviewer where necessary. The same screening process will be applied to the abstracts and full texts that are only available in other languages than English that are eligible for inclusion (Spanish, French, Portuguese, Chinese, Russian, German, Dutch, Italian, and Arabic). In our team of individual reviewers at least one (but often two) reviewer(s) speaks each of these languages. When only one team member speaks the necessary language, we will invite and train additional independent reviewers from our wide pool of collaborators and colleagues.

### Quality assessment

The quality will be assessed for each of the review papers included after the full text review stage using A MeaSurement Tool to Assess Systematic Reviews (AMSTAR) 2 critical appraisal tool for systematic reviews ( Shea *et al.*, 2007). AMSTAR 2 is based on 16 assessment questions focussed on methodological quality of systematic reviews. It has been recognised as suitable for assessing the quality of systematic reviews of studies evaluating health care interventions, including randomised and non-randomised trials ( Shea *et al.*, 2017). Therefore, AMSTAR 2 is particularly suitable for our proposed umbrella review on climate change mitigation actions, which will include (although will not be limited to) studies with designs similar to those evaluating public health interventions. In addition to the AMSTAR 2 assessment criteria, we will extract information on the last search data in each review and incorporate that into the critical appraisal to identify “synthesis update gaps”, i.e., the topics and reviews that need updating based on the currency of the search dates as well as the likelihood of additional primary study evidence being available. The critical appraisal will be performed by an expert reviewer. A second reviewer will independently assess quality of a random sample of the papers. Any differences will be reviewed and reconciled, consulting a third reviewer where necessary.

For primary studies, we will extract the risk of bias rating that the study was assigned by the relevant systematic review, where the reviews report results of such appraisal. In addition, we will appraise the primary studies by classifying the directness and nature of their evidence: modelled evidence only *vs* evidence observed at the health exposure or determinant level and modelled at the human health outcome level *vs* evidence observed at the health exposure or determinant level *vs* evidence observed at the health outcome level. Studies providing observed evidence will be further classified by whether they attempted to apply an appropriate type of control for confounding for the study design. Modelling studies will be classified into studies with and without a quantitative analysis of uncertainty, as an important for element of modelled study critical appraisal ( Hess *et al.*, 2020; Remais *et al.*, 2014).

### Data extraction

The data extraction process will be first piloted on a subset of studies to ensure coherence. All data extraction will be performed by one reviewer. A second reviewer will independently extract data for a random sample of 10% of the reviews to verify accuracy of the data extraction. Should there be substantial differences in the judgement of the two reviewers, further comparisons and reconciliation will be undertaken to achieve an acceptable agreement on the extracted data and accuracy of the extracts. 

Our preliminary examination of relevant systematic reviews demonstrated that data on the impact of the actions on health and GHG emissions are often not presented in the systematic reviews but are available in the primary studies included in such reviews. Important details of the impact measures and action characteristics that are required to draw comparisons of the impact across different actions (e.g., the scale at which the action was implemented and specific GHGs that were considered in the impact estimates) are also often lacking in the systematic reviews but are available in the primary studies. To capture these data, we will extend our data extraction process beyond the data reported in our examined systematic reviews to include the data reported in those primary studies that are included in these systematic reviews which meet our primary study inclusion criteria (see the section on inclusion criteria above). Where systematic reviews present a quantitative synthesis but insufficient detail on the results of individual primary studies to allow us to re-produce their synthesis, we will contact the review authors to request the data they extracted from the primary studies.

Hence, the data extraction process will be conducted at two levels. First, data will be extracted from the included systematic reviews in EPPI-Reviewer:

(1) Citation
(2) Type of review (systematic with or without meta-analysis)
(3) Number of bibliographic databases searched
(4) Final search date (month, year)
(5) The range of publication dates of the studies included in the review
(6) Number of studies included in the review
(7) Number of study participants covered by the review (where applicable)
(8) Types of studies included in the review (modelling, experimental, and observational with or without a comparison)
(9) Counterfactual (used in modelling studies) or comparison group (in empirical studies)
(10) Level of geographical coverage of the review (global, regional, national, city, another level – specified in the review)
(11) Setting (high-, middle-, low-income, all)
(12) Type of study quality appraisal method (i.e., the risk of bias assessment tool) used by the review
(13) Overall rating of the quality of studies included in the review (overall quality, % of studies assessed as good quality)
(14) Pooled estimates of GHG reductions together with 95% confidence intervals and heterogeneity statistics (I-squared and tau-squared) (where applicable)
(15) Pooled estimates of health impacts together with 95% confidence intervals and heterogeneity statistics (I-squared and tau-squared) (where available)
(16) Any synergies and trade-offs suggested by the review authors

Second, data from the individual studies included within each review will be extracted into an MS Excel-based data extraction tool, whose preliminary structure is illustrated in Table 2.

**Table 2.** Variables for data extraction from individual (primary) studies. Acronyms: AFOLU: agriculture, forestry, and other land use; CH4: methane; CI: confidence interval; CO2: carbon dioxide; DALY: disability-adjusted life years; GHG: greenhouse gas emissions; ITS: interrupted time series; RCT: randomised controlled trial; N2O: nitrous oxide.

Data extraction variable	Notes
Authors	Of the review
Title	Of the review
Number of included studies	In the review
Number of studies relevant for the umbrella review	How many studies in the review report GHG and health impact measurements
Relevant study citation	Primary study citation
Studies appear in multiple systematic reviews of the umbrella review	Flags primary studies included in the umbrella review from more than one systematic review
Sector	Energy, transport, AFOLU, buildings, oceans, industry, human settlements, health care, education
Sub-sector	No pre-determined list—elaborate on the sector selected above
Country(-ies)	Country of study
Action	Briefly describes the mitigation action
Pathfinder categories of action	The most relevant of the following pre-defined categories: energy-efficient transportation, alternatives to private cars: public transport, active transport, cross-sectoral actions and policy instruments, lower emission energy sources, energy efficiency of buildings and appliances, improved agricultural processes (increased efficiency and reduced input use), dietary shift towards low-emission land-based products, improved industrial processes, low-emission fuels
Description	Describe the mitigation action, including any data relevant to the timeframe, methods, and treatments
Experiment	Flags a 'new' mitigation action, potential new way of doing things
Baseline	Specifies the type of comparison without the mitigation action
Modelled or implemented action	Was the action modelled or implemented in practice
Study design	E.g., RCT, ITS, cross-sectional and case-control studies, controlled before *vs* after
Uncertainty estimation	If this is a modelling study, does it report any quantitative analysis of uncertainty
Model/action scale	Scale of the model or mitigation action
Impact scale	E.g., farm, local, city, country-wide
Timeframe of action	Timeframe over which the action has been implemented or modelled
Timeframe of impact	Specify if different from the timeframe of action
GHG indicator	CH _4_, CO _2_, N _2_O (black carbon, ozone)
GHG unit	E.g., kgCO _2_
GHG measure (action)	GHG measure for the estimate with the action
Modelled or empirical GHG estimate	Was the estimate modelled or empirical
GHG lower CI	Lower CI (if available) for the action estimate
GHG higher CI	Higher CI (if available) for the action estimate
Absolute value for baseline GHGs	GHG measure for the baseline estimate
GHG lower CI	Lower CI (if available) for the baseline estimate
GHG higher CI	Higher CI (if available) for the baseline estimate
Health indicator	Particulate matter (PM), NO _x_, deaths, respiratory disease incidence, etc.
Health unit	E.g., DALYs
Health measure	Health measure for the estimate with the action
Health lower CI	Lower CI (if available) for the action estimate
Health higher CI	Higher CI (if available) for the action estimate
Modelled or empirical health estimate	Was the estimate modelled or empirical
Impact pathway	If the health measure is a health outcome, what route(-s) of exposure or health determinants were considered when estimating or modelling the health impact
Absolute value for baseline health measure	Health measure for the baseline estimate
Health lower CI	Lower CI (if available) for the baseline estimate
Health higher CI	Higher CI (if available) for the baseline estimate
Synergies / trade-offs	Effects that enhance or reduce the effectiveness of or the capacity to implement other actions
Units of change for spillover 1	E.g., DALYs
Amount of spillover	The estimate of the spillover effect
Lower CI for spillover	Lower CI (if available) for the spillover effect estimate
Upper CI for spillover	Upper CI (if available) for the spillover effect estimate
Quality	Rating of primary study by review authors
Comment	Any definitions, important notes, abbreviations, elaborations of standard practice, etc.

### Synthesis

To determine what types of climate change mitigation actions with defined health co-benefits have been reported in systematic reviews (research question 1), we will use narrative/thematic synthesis developing a typology of the reported actions. We will further use Sankey diagrams to map the pathways (i.e., routes of exposure and health determinants) through which the health impact of the identified actions in each sector and sub-sector is estimated. The pathway maps will demonstrate what exposure routes and GHG and health outcomes have been linked in existing research and how. We will also narratively synthesise any other effects of our identified actions, e.g., any synergies (i.e., effects of actions that apart from their own impact on GHG emissions and health co-benefits also enhance the effectiveness of other climate change mitigation actions of the capacity to implement the other actions) and trade-offs (i.e., effects of a climate mitigation action that preclude implementation of other climate mitigation actions or minimise the effectiveness of the other actions) suggested by the review authors, potential for unintended consequences, spillover effects, and distributional effects with implications for health equity.

Particular attention will be paid to the timeframe over which actions are shown to exert effects on health and GHG emissions/concentrations and location where the health effects are experienced (e.g., locally, regionally, globally).

To compare how the magnitude of change in health outcomes or exposures and GHG emissions or concentrations impact differ within the identified types of actions, based on the modelled as compared to the observational evidence and across different modelling and implementation contexts (research question 2), we will perform quantitative syntheses of the impact of the reviewed actions on health outcomes and GHG emissions, where possible, provided sufficient homogeneity across the individual effect estimates. If pooling of findings across studies is feasible for any particular outcome, inverse-variance weighted random effects meta-analysis will be used. Meta-regression models may also be estimated to explore heterogeneity across outcomes by types of actions or exposures or contextual factors. We propose to use the standard methods of synthesis to pool findings across studies should enough data be available. This might incorporate inverse-variance random effects meta-analysis for particular mitigation strategies and outcome constructs, where measures of the variance (or variables closely relating to it, such as sample size) are presented and appropriate. Another option is to conduct meta-regression analysis (MRA), which enables pooling of effects across mitigation strategies and outcome constructs in a larger model. Where this is possible, we will use appropriate methods of weighting depending on the underlying literature (empirical literature might use inverse-variance, modelling literature might use population measures). In producing any quantitative syntheses, we will take account of any studies that are overlapping, i.e., captured by multiple included systematic reviews, by only including one estimate per primary study for each outcome synthesised. Where quantitative syntheses are not appropriate, evidence of effects on health, its well-established determinants, and GHG emissions will be synthesised narratively. Our synthesis of the effects and quality of the evidence will be stratified by sector, action and health outcome type, modelled *vs* observational evidence, different geographical modelling and implementation contexts, and, where possible, any evidence of implications for health equity. The direction and strength of effect and quality of evidence (including effect heterogeneity across the reviews) will be reported and illustrated graphically for each stratum, with relevant graphs and figures presented where possible, for example using forest plots.

To determine how syntheses differ in their coverage, quality, and strength of evidence across sectors, disciplines, action types, and their implementation or modelling contexts (research question 3), we will use the EPPI Mapper tool to produce visual maps that present the evidence as a matrix classifying the evidence by the characteristics of interest. The maps will help identifying the key gaps in the available evidence syntheses, particularly in relation to different geographies, contexts, sectors, and types of actions. We will map the ‘absolute gaps’ in evidence that require new primary studies, ‘synthesis gaps’ that require new focussed systematic reviews and syntheses, and ‘update gaps’ where existing reviews are based on outdated searches and updates of the existing focussed reviews are necessary. 

## Discussion

This paper presented the protocol for an umbrella review of the systematically synthesised evidence on climate change mitigation actions of benefit for health. To our knowledge, this will be the first attempt at an integrated synthesis of actions examined in systematic reviews across all sectors and disciplines that can help lead to system wide transitions that are necessary for the net zero carbon future.

We acknowledge that our proposed review is subject to a number of potential sources of bias:

1. **Inconsistent definitions** may be used across systematic reviews, particularly when those pertain to different sectors or originate from different disciplines or research communities. To minimise this bias, we will set our own broad definitions for key terms accounting for different disciplinary and sectoral perspectives and follow those in a consistent manner throughout our evidence synthesis, reporting, and communication of our results. Feedback will be sought from the multi-disciplinary panel of commissioners and partners of the Pathfinder Initiative, where necessary.
2. **Risk of bias in the effect size calculations** can arise as different primary papers and systematic reviews may use different formulas to calculate their examined effect sizes for the health and GHG emission impact of the actions. To address this, we will translate between the different effect size types, where possible, using established methods such as on converting between standardised mean difference and odds ratios ( Deeks *et al.*, 2021). Suggestions for specialised translation methods will be sought from the panel of commissioners and partners of the Pathfinder Initiative, where necessary.
3. **Reviewer bias** can arise when evaluating records retrieved from the searches for inclusion and exclusion as a result of differences in the way reviewers interpret the inclusion and exclusion criteria. To minimise the risk of such bias, we will train all reviewers to apply the inclusion/exclusion criteria in a consistent manner. The training will be done on an initial sample of studies that all reviewers will first individually assess for inclusion and then collectively reconcile in consultation engaging in an in-depth critical discussion of any differences in their judgement. For the rest of the screening process, each record will be reviewed by a pair of reviewers and any differences in judgement will be reconciled, referring to a third reviewer, where necessary. Reviewer bias can also be introduced in data extraction. To minimise the risk of such bias, data extraction will be first piloted for a small sample of studies with extracts reviewed and their accuracy agreed with other team members before the rest of the data extraction process takes place. A sample of all data extracts will be reviewed by a second reviewer with any differences reconciled involving a third reviewer, where necessary, and expanding the sample, if required.
4. **Publication bias** may arise for four reasons: (1) more original studies of climate change mitigation actions are conducted and subsequently synthesised in systematic reviews in high income settings than low-income settings, which would introduce an unavoidable geographical location bias; however, higher income countries also have higher per capita GHG emissions and mitigation is therefore a priority for these countries. (2) more systematic reviews may have a focus on developed and high-income countries (or vice versa) and their searches may therefore be restricted to high income countries (or vice versa) (3) Studies published in English – as the "prima lingua" of the modern science – are more likely to be included in systematic reviews than studies published in other languages, which introduces a language bias; although we include systematic reviews published in other languages, and the critical appraisal done under AMSTAR 2 assesses whether systematic reviews published in English are themselves limited to English language studies only. (4) Studies demonstrating evidence for an effect of an action on the outcome of interest are more likely to be published than studies that are not able to demonstrate evidence of effect (such as due to insufficient statistical power), subsequently introducing bias towards capturing actions with demonstrated impacts as opposed to without; we attempt to address this by incorporating primary studies in synthesis regardless of publication status, and the critical appraisal under AMSTAR 2 also assesses whether formal publication bias tests have been done in the review. (5) Systematic reviews are more prevalent in the disciplines with a strong tradition of this approach to evidence synthesis, e.g., medicine, public health, and criminology, than others, e.g., global environmental change and development studies, where evidence derivation and synthesis tradition is rooted in differently, and often less rigorously, structured assessment and summary reports ( Minx *et al.*, 2019; Tusting *et al.*, 2021; Waddington *et al.*, 2012). Our umbrella review focuses on actions with documented impact on health and its well-established determinants, and incorporates evidence from two fields where concerted efforts are being made to improve the quality of primary studies and evidence synthesis (environmental science and development studies).

As with all umbrella reviews, our proposed review can only synthesise what other researchers have investigated, published, and systematically reviewed ( Fusar-Poli & Radua, 2018). We foresee that the systematic reviews that our umbrella review will cover will likely lack quantitative syntheses, as we have seen in our preliminary examination of relevant systematic reviews. To address this challenge, we will extend our data extraction to primary studies to produce our own quantitative syntheses and meet our research objectives. This leads to another potential source of bias, as our umbrella review will not be covering any primary studies that are not captured by the identified systematic reviews. Hence, we will not capture primary studies that are not included by these syntheses, e.g., because they were published after the date when the literature searches for the reviews were conducted, and primary studies in areas where systematic reviews have not been conducted. To account for these gaps in our syntheses, we will record the date of the last search date of each review and use it with other characteristics of the evidence to examine the ‘synthesis gaps’ and ‘synthesis update gaps’, as explained earlier.

Another foreseeable challenge is the likely heterogeneity across the primary studies, which may limit the extent to which we will be able to perform quantitative syntheses of their results for some of the actions. For example, Hess *et al.* (2020) reported that studies of health and GHG impacts of climate change mitigation actions often use different metrics of success and timescales, and often have limited reporting of baseline health outcome or exposure and GHG emission levels. We will endeavour to examine the sources of heterogeneity and account for uncertainly in any quantitative syntheses that we will perform.

This umbrella review is intended to provide a robust synthesis of the nature and quality of the published and systematically synthesised evidence on actions for climate change mitigation and health. It will complement other research components of the Pathfinder Initiative, which will synthesise evidence from cases where climate change mitigation actions have been implemented in different contexts in practice. In the future, this umbrella review could be turned into a living review to help speed up the incorporation of the latest evidence into climate change mitigation policy and practice in a manner that allows simultaneously achieving the greatest health benefits ( Sethi *et al.*, 2020).

## Conclusion

This protocol presents our approach to the development of an umbrella review of actions for climate change mitigation and health. It will provide a robust and transparent assessment of the quality of the available evidence for the actions and their effects reported in published systematic reviews across a wide range of disciplines and sectors. The review will help identifying the most effective actions, as indicated by the published evidence syntheses, and gaps in the evidence syntheses.

## Ethics, outputs, and dissemination

As a project entirely comprised of synthesis of existing evidence and not involving any individual participants or patients, the study does not require ethical approval. However, the study protocol has undergone consultation with the Observational Ethics Committee of the London School of Hygiene & Tropical Medicine.

Results of the umbrella review will be presented in a peer-reviewed academic paper. Furthermore, key messages from the review will be communicated thorough the Lancet Pathfinder Commission report which will be published in late 2022, at relevant international meetings, and through Pathfinder Initiative partner organisations (OECD, SDSN, C40 Cities, CDP and the Alliance for Health Policy and Systems Research), using targeted policy briefs appropriate to each partner's network.

## Study status

The literature searches and screening for inclusion have been completed. Data extraction is in progress.

## Data availability

### Underlying data

No underlying data are associated with this article.

### Extended data

LSHTM Data Compass: Search strategy for the umbrella review "Quantifying the effectiveness and health co-benefits of climate change mitigation actions across sectors: a protocol for an umbrella review". https://doi.org/10.17037/DATA.00002761 ( Belesova *et al.*, 2022)

Data are available under the terms of the Creative Commons Attribution 3.0 Unported (CC-BY 3.0).
