# Supplementary material for: Quantifying the effectiveness and health co-benefits of climate change mitigation actions across sectors: a protocol for an umbrella review
Source: Wellcome Open Res. 2022 Aug 25;7:98. Originally published 2022 Mar 18. [Version 2] doi: 10.12688/wellcomeopenres.17498.2 (PMC10333776; doi:10.12688/wellcomeopenres.17498.2)
Supplement: Peer review history

# Peer review: Reviewer response for version 2

**DOI:** 10.21956/wellcomeopenres.20134.r58589

**Published:** 2023-07-13

**Authors:** Jesse E Bell, Kelli Gribben

**Affiliations:** 1 University of Nebraska-Lincoln, Lincoln, Nebraska, USA; 2 University of Nebraska Medical Center, Omaha, NE, USA

The reviewer thinks this is an important effort and thank the authors and the journal for the opportunity to review this protocol. I've provided my comments and decision below.

 Summary

 Publication describes a protocol for conducting an umbrella review on climate change mitigation actions inclusive of multiple sectors (Energy systems, agriculture, forest, and other land use, transport, buildings, industry, and land coastal and ocean sinks, and engineered sinks), environmental indicators, health measures, and geography (local to global). The study aligns with the goal of the Pathfinder Initiative by applying a broad inclusion criteria to capture all available evidence from peer-reviewed systematic reviews to comprehensively evaluate and estimate the impacts of climate change mitigation activities on health and identify gaps.

 Introduction

 Rationale and research questions – clearly stated on page 3. The proposed umbrella review will expand on previous evidence from individual systematic reviews and meta-analyses by considering cross-sectoral, cross disciplinary, and system-wide implications of climate change mitigation actions on health. This will be the most comprehensive review and meta-analysis of climate change mitigation and health to direct future activities by diverse stakeholders.

 Protocol

 Inclusion criteria

 Population – I wonder if the authors can be more specific here? page 4 under Protocol inclusion criteria. Suggested change/example:

 Populations: Published systematic reviews, meta-analyses, narratives on climate change mitigation interventions or predictive analyses related to climate indicators and human health measured on any geographical scale (local to global) year 2000-[date].

 Discussion

 There is a need to clarify the stratification of evidence plan on page 9. From the text: “Our synthesis of the effects and quality of the evidence will be stratified by sector, action and health outcome type, modelled vs observational evidence, different geographical modelling and implementation contexts, and, where possible, any evidence of implications for health equity. The direction and strength of effect and quality of evidence (including effect heterogeneity across the reviews) will be reported and illustrated graphically for each stratum, with relevant graphs and figures presented where possible, for example using forest plots.”

 Do the authors intend to grade the evidence for each stated stratum based on the quality of evidence? If so, what will the criteria be for each level of evidence?

 What software will be used for the meta-analyses?

 Can the authors provide more detail on any potential sensitivity analyses?

 Is there a plan to describe limitations of the umbrella review in detail? This can provide much needed context for the results.

Is the study design appropriate for the research question?

Yes

Is the rationale for, and objectives of, the study clearly described?

Yes

Are sufficient details of the methods provided to allow replication by others?

Partly

Are the datasets clearly presented in a useable and accessible format?

Not applicable

Reviewer Expertise:

climate and respiratory health

We confirm that we have read this submission and believe that we have an appropriate level of expertise to confirm that it is of an acceptable scientific standard, however we have significant reservations, as outlined above.

# Peer review: Reviewer response for version 2

**DOI:** 10.21956/wellcomeopenres.20134.r58584

**Published:** 2023-07-10

**Authors:** Mirko S Winkler

**Affiliations:** 1 University of Basel, Basel, Switzerland

Congratulations to the authors for this well-developed high-quality study protocol.

 Here my few observations:

 A) Is my understanding correct that, in a simplified manner, you will follow the following steps for conducting your umbrella review: 1) You will, through your literature search, identify “systematic reviews or meta-analyses”. 2) You will then extract primary studies from the identified systematic reviews or meta-analyses based on your criteria. 3) Those primary studies will then be characterized and synthesized. If my understanding is wrong, I am inviting the authors to further clarify the overall process. E.g. An overview of the main steps could be provided early in the methods chapter. In any case, it would be nice to have a simple graphic illustrating the overall process of the umbrella review.

 B) The authors state that they will map gaps in existing evidence that require new primary studies. Will this assessment of gaps only be done based on the primary papers identified in the systematic reviews/meta-analyses or does it also reflect on health co-benefits that have not yet been studied/been included in systematic reviews/meta-analyses? This should be clarified, including methodological aspects.

 Minor observations:

1. Search term: will you apply the search term in different languages? Seems not yet clear to me.
2. The following sentence is hard to read (a dash seems missing): “Here we report a protocol for an umbrella review – a review of systematic reviews or meta-analyses, undertaken as a part of the Pathfinder Initiative, to synthesize the existing syntheses of findings on the range and effectiveness of actions – both potential and already implemented in practice – for climate change mitigation and health across all sectors and disciplines that can help triggering and sustaining the necessary system-wide transitions.”
3. Suggesting to also include “well-being” as a health outcome, as mental health does not fully capture the well-being dimension.
4. Suggesting to also include antimicrobial resistance as a risk factor, as it is linked to health and climate change (e.g. increased temperatures increase both the rate of bacterial growth and the rate of the spread of antibiotic-resistant genes between microorganisms).
5. Since your review is about “health co-benefits”, shouldn’t you also include health promoters (e.g. spending more time outdoors, increased physical activity, fewer cold days (in the north, etc.) in addition to health risks?

Is the study design appropriate for the research question?

Yes

Is the rationale for, and objectives of, the study clearly described?

Yes

Are sufficient details of the methods provided to allow replication by others?

Yes

Are the datasets clearly presented in a useable and accessible format?

Yes

Reviewer Expertise:

Urban public health, health impact assessment, epidemiology

I confirm that I have read this submission and believe that I have an appropriate level of expertise to confirm that it is of an acceptable scientific standard.

# Peer review: Reviewer response for version 2

**DOI:** 10.21956/wellcomeopenres.20134.r58590

**Published:** 2023-07-10

**Authors:** Martin W Bloem

**Affiliations:** 1 Johns Hopkins University, Baltimore, Maryland, USA

The proposal by Belesova et al. presents a comprehensive "umbrella review" that systematically examines quantitative studies on the health co-benefits of climate change mitigation policies. The primary objective of this research is to advance our understanding of the magnitude and significance of the health impacts associated with actions taken to address climate change. By synthesizing the existing literature, this analysis aims to provide valuable insights and inform policy discussions in the context of the global climate crisis.

 The authors have already acknowledged the concern raised by Belle Workman and Kathryn Bowen regarding the potential bias towards developed economy countries in the published systematic literature base. Although the authors have taken steps to address this issue through adjusted methodology, it is important to highlight two additional points:

 Firstly, the observation that a greater number of original studies on climate change mitigation actions are conducted and synthesized in high-income settings compared to low-income settings highlights an important aspect of the current research landscape. While it is true that higher-income countries have higher per capita greenhouse gas (GHG) emissions, and thus mitigation is a crucial focus for these countries, it is equally important to recognize the transboundary nature of GHG emissions and their impacts. Research conducted by organizations such as Oxfam has demonstrated the disproportionate impact of climate change on low- and middle-income countries (LMICs) (OXFAM, 2022) ^1^. Despite contributing a minimal percentage of global carbon emissions, these countries have experienced escalating hunger crises, heat waves, and other extreme weather events in climate hotspots. Therefore, it is imperative to acknowledge and address the geographical bias in climate change mitigation studies. By doing so, we can address the interconnected and unequal distribution of climate change impacts and gain insights into the unique challenges faced by LMICs.

 Secondly, it is important to note that the study utilizes modeling studies, which have inherent limitations, particularly when it comes to capturing the complexities and unique health challenges of LMICs. Global modeling studies that examine the consequences of climate action on health rely on data and assumptions that may not fully capture the nuances of LMICs. Consequently, there is a risk of underestimating the impact of climate action on morbidity, mortality, and nutritional requirements in LMICs.

 For instance, the EAT-Lancet diet, developed through global modeling, presents differential impacts for LMICs. While this diet aligns with GHG targets in line with the Paris Agreement and has the potential to reduce emissions by up to 80%, a study by Semba et al. demonstrated that in predominantly LMICs, agricultural GHG emissions would increase by 12-283%. This is due to the nutritional requirements in LMICs, where animal-source foods are still essential to ensure dietary adequacy (Semba et al., 2020; Willett et al., 2019) ^2^^,^^3^.

 This finding is also reflected in the critique of Low- and Middle-Income Countries (LMICs) at COP meetings revolving around their challenge of balancing economic development priorities with the disproportionate impacts of climate change, despite their minimal contribution to global greenhouse gas emissions. Important research like these types of studies should include these facts in their discussion.

Is the study design appropriate for the research question?

Yes

Is the rationale for, and objectives of, the study clearly described?

Yes

Are sufficient details of the methods provided to allow replication by others?

Yes

Are the datasets clearly presented in a useable and accessible format?

Yes

Reviewer Expertise:

Nutrition, Public Health, NCDs, Climate Change, Policies, Micronutrients, Humanitarian, Emergencies, HIV/AIDS, Infectious diseases.

I confirm that I have read this submission and believe that I have an appropriate level of expertise to confirm that it is of an acceptable scientific standard.

## References

[1] : Hunger in a Heating World, How the climate crisis is fuelling hunger in an already hungry world.2022; Reference source

[2] : Adoption of the 'planetary health diet' has different impacts on countries' greenhouse gas emissions. *Nat Food* .2020;1(8) : 10.1038/s43016-020-0128-4 481-484 10.1038/s43016-020-0128-4 37128073

[3] : Food in the Anthropocene: the EAT–Lancet Commission on healthy diets from sustainable food systems. *The Lancet* .2019;393(10170) : 10.1016/S0140-6736(18)31788-4 447-492 10.1016/S0140-6736(18)31788-4 30660336

# Peer review: Reviewer response for version 2

**DOI:** 10.21956/wellcomeopenres.20134.r52171

**Published:** 2022-10-13

**Authors:** Jonathan A. Patz

**Affiliations:** 1 Department of Population Health Sciences, University of Wisconsin-Madison, Madison, WI, USA

I've read through the responses and the revised manuscript, and I approve since the article is scientifically valid in its revised form.

Is the study design appropriate for the research question?

Yes

Is the rationale for, and objectives of, the study clearly described?

Yes

Are sufficient details of the methods provided to allow replication by others?

Yes

Are the datasets clearly presented in a useable and accessible format?

Yes

Reviewer Expertise:

Environmental public health, climate change and health

I confirm that I have read this submission and believe that I have an appropriate level of expertise to confirm that it is of an acceptable scientific standard.

# Peer review: Reviewer response for version 1

**DOI:** 10.21956/wellcomeopenres.19348.r49304

**Published:** 2022-05-30

**Authors:** Kathryn Bowen, Belle Workman

**Affiliations:** 1 Melbourne Climate Futures, Melbourne School of Population and Global Health, University of Melbourne, Melbourne, Australia; 2 Melbourne Climate Futures, University of Melbourne, Melbourne, Australia

This paper is a helpful step to better understand mitigation actions and their health effects. However, a large caveat is that this is based on the published systematic literature base, which has a bias towards developed economy countries. Suggestions are provided to strengthen the paper, including clarity on the health outcomes of interest, and a stronger focus on determinants of health (e.g. governance). Detailed comments can be found in the following annotated manuscript pdf: *https://s3-eu-west-1.amazonaws.com/wellcomeopenresearch/linked/266422.Belesova_et_al_AW%2BKBreview.pdf*

Is the study design appropriate for the research question?

Yes

Is the rationale for, and objectives of, the study clearly described?

Yes

Are sufficient details of the methods provided to allow replication by others?

Yes

Are the datasets clearly presented in a useable and accessible format?

Not applicable

Reviewer Expertise:

NA

We confirm that we have read this submission and believe that we have an appropriate level of expertise to confirm that it is of an acceptable scientific standard, however we have significant reservations, as outlined above.

# Response

**Published:** 2022-07-31

**Authors:** Kristine Belesova

**Affiliations:** LSHTM, UK

*1. A large caveat is that this is based on the published systematic literature base, which has a bias towards developed economy countries*
  This indeed is a potential bias and we have now further elaborated on it in the discussion section of the protocol on page 17. However, having by now completed screening and classification of the included systematic reviews, we are finding a good balance in systematic review focus on developing and developed economies. Most of the geographical bias would be originating from the higher proportion of original studies focussing on developed economies. Our evidence mapping will be illustrating the geographical balance of both systematic reviews and original studies.
*To expand the last sentence of the first introductory paragraph by adding “and avoid adversely affecting health outcomes (e.g., through regressive policies)”*
  We expanded the sentence as suggested on page 4.
*To briefly contextualise the scope of the initiative here by addressing why adaptation is out of scope, given that adaptation policy is often where health is incorporated*
  We have added the suggested edits on page 4.
*The IPCC uses four slightly different system transitions - would be good to adapt this for consistency, unless there is a good reason not to.*
  To our knowledge, the IPCC has two different categorisations of system transitions: one in AR6 WG3 focussed on mitigation ( https://www.ipcc.ch/report/ar6/wg3/figures/summary-for-policymakers/figure-spm-8/) and another in the AR6 WG2 focussed on Impacts, Vulnerability, and Adaptation ( https://www.ipcc.ch/report/ar6/wg2/figures/summary-for-policymakers/figure-spm-4/).
  The system transitions we illustrated in Table 1 were mainly focussed on the IPCC AR6 WG3 classification, as it was more closely aligned with the focus of our umbrella review on mitigation. However, although the categories used in the IPCC AR6 WG3 report summarised the IPCC evidence-base well, we are finding that this framework is not encapsulating the results that we are obtaining from the umbrella review. As such, we are considering other classifications, such as the classification used in Project Drawdown. Currently, the Drawdown Framework for Climate Solutions seems to be better suited to our data as it includes specific solutions, which are grouped into broader areas of action within the different sectors. This framework offers clearer boundaries on where individual actions can be placed. However, any classification we use will be inevitably adapted in view of our results. Therefore, we are also considering ways in which to adapt the Drawdown Framework to also reflect system transitions and cross-cutting actions. As such, we have removed the table from the manuscript, and instead, we have now elaborated (on page 6) that we are considering several classification systems and will choose and adapt the one that best fits our data to present our findings.
*To explain the meaning of the shaded cells in Table 1.*
  The shading had no meaning, it must have been introduced by accident while formatting the table. We have removed the table from the manuscript, as explained above.
*Edit the Table 5 in response following the specific suggestions made on selected lines of the table*
  We have made the suggested specific edits on pages 7 and 8. We would like to refrain from broader revisions of the list of risk factors, as it is taken directly from the Global Burden of Disease (GBD) project. We have now added a reference to the GBD for clarity.
*To elaborate how the abstracts and full texts identified in other languages will be screened.*
  We have now elaborated on this on page 10.

# Peer review: Reviewer response for version 1

**DOI:** 10.21956/wellcomeopenres.19348.r49305

**Published:** 2022-04-04

**Authors:** Jonathan A. Patz

**Affiliations:** 1 Department of Population Health Sciences, University of Wisconsin-Madison, Madison, WI, USA

This manuscript describes a cross-sectoral “umbrella review” of quantitative studies on the health co-benefits of climate change mitigation policies.  Such information is critical in advancing our understanding of the magnitude of health benefits (or disbenefits) of actions taken to avert climate change.  This analysis, therefore, will be important and timely in the policy discourse around the global climate crisis.

 The methods are appropriate and thorough. The approach to the review, however, can be clarified especially related to systematic reviews versus primary studies.  For example, while the authors stipulate that they intend to provide a comprehensive synthesis across published systematic reviews, in their preliminary analysis, they already state that key information (that may exist within a primary study) does not appear in the systematic review in which that study appears.  So the authors state, “...we will extend our data extraction to primary studies to produce our own quantitative synthesis.”   How this selection of primary studies needs to be clarified, and it also calls to question why the team’s approach limits its efforts to only include studies that have been captured within the (relatively few) systematic reviews, knowing the deficiencies already discovered. 

 Also, additional methodological description of “synergies and trade-offs” would be useful. Mapping pathways of benefits across sectors is important and the authors specify an “inverse-variance weighted random effects meta-analysis”. I think an example and further explaining this approach is warranted.  Further, I’d like to know how this approach is similar or different from optimization modelling.

Is the study design appropriate for the research question?

Yes

Is the rationale for, and objectives of, the study clearly described?

Yes

Are sufficient details of the methods provided to allow replication by others?

Yes

Are the datasets clearly presented in a useable and accessible format?

Yes

Reviewer Expertise:

Environmental public health, climate change and health

I confirm that I have read this submission and believe that I have an appropriate level of expertise to confirm that it is of an acceptable scientific standard, however I have significant reservations, as outlined above.

# Response

**Published:** 2022-07-31

**Authors:** Kristine Belesova

**Affiliations:** LSHTM, UK

1. *The approach to the review, however, can be clarified especially related to systematic reviews versus primary studies.  For example, while the authors stipulate that they intend to provide a comprehensive synthesis across published systematic reviews, in their preliminary analysis, they already state that key information (that may exist within a primary study) does not appear in the systematic review in which that study appears.  So the authors state, “...we will extend our data extraction to primary studies to produce our own quantitative synthesis.”   How this selection of primary studies needs to be clarified, and it also calls to question why the team’s approach limits its efforts to only include studies that have been captured within the (relatively few) systematic reviews, knowing the deficiencies already discovered. *
  Our ambition is to undertake an umbrella review and produce a comprehensive comparative meta-synthesis of the evidence that has been captured by existing systematic reviews. However, after reviewing some of the systematic reviews that were eligible for inclusion in our analysis, we concluded that the data that is reported in the systematic reviews often falls short from the level of detail that is required for our analyses. For example, many systematic reviews are narrative and do not attempt any quantitative synthesis even when such synthesis would be feasible. In some cases, this coincides with poor quality of the review; in others with their focus on research and synthesis questions that do not necessarily require a quantitative synthesis. Furthermore, systematic reviews without a quantitative synthesis often do not report sufficient data from the primary studies that they reviewed, e.g., only reporting either the health or GHG impact of actions but not both or lacking information on the baseline or scale of the impact, which are necessary to allow comparing the impact of actions across studies and settings. However, these data are often available from the text of the primary studies. Therefore, we adapted the umbrella review methodology to expand our data extraction procedure beyond the (often limited) text of the systematic reviews to the text of primary studies that were synthesised in the systematic reviews that met our inclusion criteria. In other words, we are referring to the primary studies to overcome the limited (and sometimes poor quality) of reporting by the systematic reviews.
  Primary studies that have not been captured in existing systematic reviews were beyond the scope of our synthesis. Their inclusion would not be justifiable as a part of the umbrella review methodology and would require a fundamentally different approach to literature identification at the outset targeting primary studies instead of systematic reviews. Such an approach is beyond the scope of our project and resources. Hence, our choice of the umbrella review methodology was largely pragmatic – aiming to produce a meta-synthesis of the evidence that has been captured in existing systematic reviews. Through our umbrella review, we also aim to map the gaps in existing systematic reviews, indicating areas and topics where systematic reviews are lacking or have not been recently updated. We will be highlighting these gaps in our results making recommendations for future systematic reviews and updates of existing systematic reviews.  
  We have now elaborated these points in the text of our protocol on page 8. We also have now defined the term “umbrella review” on pages 4-5.
2. *Additional methodological description of “synergies and trade-offs” would be useful*
  We will extract any information on synergies and trade-offs from the systematic reviews, as it is presented by the authors of the systematic reviews. As synergies we consider the effects of actions that, apart from their own impact on GHG emissions and health co-benefits, also enhance the effectiveness of other climate change mitigations actions or the capacity for other actions to be implemented. As trade-offs we consider the effects of a climate mitigation action that precludes the implementation of other climate mitigation actions or minimises their effectiveness.
  We have now elaborated these methodological aspects on page 15 of the protocol.
3. *Mapping pathways of benefits across sectors is important and the authors specify an “inverse-variance weighted random effects meta-analysis”. I think an example and further explaining this approach is warranted.  Further, I’d like to know how this approach is similar or different from optimization modelling.*
  The purpose of the mapping pathways is to demonstrate what exposure routes and GHG and health outcomes have been linked in existing research and how. This will be done using Sankey diagrams.
  A separate issue is how the evidence is synthesised. We propose to use the standard methods of synthesis to pool findings across studies should enough data be available. This might incorporate inverse-variance random effects meta-analysis for particular mitigation strategies and outcome constructs, where measures of the variance (or variables closely relating to it, such as sample size) are presented and appropriate. Another option is to conduct meta-regression analysis (MRA), which enables pooling of effects across mitigation strategies and outcome constructs in a larger model. Where this is possible, we will use appropriate methods of weighting depending on the underlying literature (empirical literature might use inverse-variance, modelling literature might use population measures).
  We have elaborated these points on pages 15-16.
